# Enhancing the estimation of PaCO_2_ from etCO_2_ during ventilation through non-invasive parameters in the ovine model

**DOI:** 10.1186/s12938-024-01292-2

**Published:** 2024-10-24

**Authors:** Mike Grüne, Lena Olivier, Valerie Pfannschmidt, Matthias Hütten, Thorsten Orlikowsky, Andre Stollenwerk, Mark Schoberer

**Affiliations:** 1 Department of Paediatric and Adolescent Medicine, RWTH Aachen University Hospital, Aachen, Germany; 2 Embedded Software - Informatik 11, RWTH Aachen University, Aachen, Germany; 3 MosaKids Children’s Hospital, Maastricht University Medical Center, Maastricht, The Netherlands

**Keywords:** Closed loop control, EtCO_2_, Mechanical ventilation, Neonates, PaCO_2_, Robust linear regression, Ventilation

## Abstract

**Background:**

In mechanically ventilated neonates, the arterial partial pressure of $\text {CO}_{2}$ ($\text {PaCO}_{2}$) is an important indicator for the adequacy of ventilation settings. Determining the $\text {PaCO}_{2}$ is commonly done using invasive blood gas analyses, which constitute risks for neonates and are typically only available infrequently. An accurate, reliable, and continuous estimation of $\text {PaCO}_{2}$ is of high interest for medical staff, giving the possibility of a closer monitoring and faster reactions to changes. We aim to present a non-invasive estimation method for $\text {PaCO}_{2}$ in neonates on the basis of end-tidal $\text {CO}_{2}$ ($\text {etCO}_{2}$) with inclusion of different physiological and ventilation parameters. The estimation method should be more accurate than an estimation by unaltered $\text {etCO}_{2}$ measurements with regard to the mean absolute error and the standard deviation.

**Methods:**

Secondary data from 51 preterm lambs are used, due to its high comparability to preterm human data. We utilize robust linear regression on 863 $\text {PaCO}_{2}$ measurements below or equal to 75 mmHg from the first day of life. $\text {etCO}_{2}$ along with a set of ventilation settings and measurements as well as vital parameters are included in the regression. Included independent variables are chosen iteratively by highest Pearson correlation to the remaining estimation deviation.

**Results:**

The evaluation is carried out on 12 additional neonatal lambs with 246 $\text {PaCO}_{2}$ measurements below or equal to 75 mmHg from the first two days of life. The estimation method shows a mean absolute error of 3.80 mmHg with a 4.92 mmHg standard deviation of differences and a standard error of 0.31 mmHg in comparison to measured $\text {PaCO}_{2}$ by blood gas analysis.

**Conclusions:**

The estimation of $\text {PaCO}_{2}$ by the proposed equation is less biased than unaltered $\text {etCO}_{2}$. The usage of this method in clinical practice or in applications like the automation of ventilation needs further investigation.

## Introduction

Respiratory failure is a common complication of preterm birth [1]. Especially those children born before the end of the 28th week of gestation, defined as extremely preterm infants by the WHO, frequently require support by mechanical ventilation [2–4]. Ventilation settings must be continuously adapted to patient needs, close monitoring of parameters of gas exchange is therefore necessary [5].

Knowledge of the arterial partial pressure of $\text {CO}_{2}$ (${\text {PaCO}_{2}}$) is essential for the management of patients with respiratory disease or respiratory failure [6]. During mechanical ventilation, knowledge about the ongoing $\text {CO}_{2}$ elimination is desired at all times to appropriately regulate minute ventilation. In clinical practice, $\text {PaCO}_{2}$ is intermittently determined by blood gas analyses [7], which require blood sampling. Especially in neonatal patients, this causes relevant blood loss and, if performed by capillary punctures, is a stressful procedure [8]. Thus, to reduce the frequency of blood sampling and to provide continuous information on $\text {CO}_{2}$ elimination in mechanically ventilated patients, a reliable, online estimation of $\text {PaCO}_{2}$ is important [9]. Two monitoring modalities are currently used for this purpose: capnometry and transcutaneous measurement of $\text {PaCO}_{2}$.

Transcutaneous measurement of $\text {CO}_{2}$ ($\text {TcpCO}_{2}$) is commonly used for continuous estimation in neonates. However, the accuracy of the method is affected by sensor-drift, skin thickness, skin temperature, and capillary perfusion [10–12]. Regular sensor calibrations are indispensable. Furthermore, $\text {TcpCO}_{2}$ requires heating of the measurement electrode to temperatures above body temperature, which can cause burns [13]. To avoid this, regular changes of the electrode position are necessary, which are dependent on the positioning of the child.

Capnometry allows the breath-by-breath monitoring of the $\text {CO}_{2}$ proportion in the respiratory gas [14]. The partial pressure of $\text {CO}_{2}$ calculated from this ($\text {etCO}_{2}$) can be used to estimate the $\text {PaCO}_{2}$ [15]. Several publications cover the correlation between $\text {etCO}_{2}$ and $\text {PaCO}_{2}$ in the paediatric and neonatal setting [16–21]. In neonates without severe lung disease, the measurements are also shown to have better reliability, accuracy and precision with $\text {PaCO}_{2}$ than $\text {TcpCO}_{2}$ [22]. The usage of $\text {etCO}_{2}$ measurements for the estimation of $\text {PaCO}_{2}$ in different settings and populations has been attempted with varying results [23–27]. The estimation of $\text {PaCO}_{2}$ from $\text {etCO}_{2}$ can be distorted by measurements of dead-space gas fractions, which did not take part in alveolar gas exchange. This is particularly valid in neonates, who have a relatively higher anatomical dead space and more importantly relatively much higher apparatus dead spaces [28]. Another disruptive factor in the measurement is intrapulmonary shunt when pulmonary capillary blood passes non-ventilated alveoli [29]. Hence, the estimation of such influences by non-invasive parameters is desirable to achieve a more accurate estimation of $\text {PaCO}_{2}$.

Using a neonatal ovine model, we previously demonstrated the feasibility of closed-loop control of $\text {PaCO}_{2}$ [30]. However, we identified a need for a more accurate estimation of $\text {PaCO}_{2}$. Our hypothesis is that the consideration of ventilation and physiological parameters results in an improved accuracy for $\text {PaCO}_{2}$ estimation and a higher individualization of the estimation in comparison to unaltered $\text {etCO}_{2}$. This should be reflected in a lower mean absolute error (MAE) and a lower standard deviation (SD) than the estimation by unaltered $\text {etCO}_{2}$. The underlying expectation is that ventilation and physiological parameters are able to estimate the different physiological, technical, and further reasons for the mismatch of $\text {etCO}_{2}$ and $\text {PaCO}_{2}$ and thus provide a more individualized estimation for applications like physiological closed-loop control (PCLC) without the need for parametrization.

## Results

### Model development

The iterative regression approach, used on the design population described in section "Population", resulted in the inclusion of four different variables in addition to $\text {etCO}_{2}$. The highest correlation to the deviation of $\text {PaCO}_{2}$ and $\text {etCO}_{2}$ was present in ${{\text {O}_{2}}{^{\text {diff}}}^2}$. As this parameter describes the difference between the set ${\text {FiO}_{2}}$ and the measured ${\text {SpO}_{2}}$, it is directly dependent on the current ventilation parameters. The remaining considered variables are ${\%_{\text {spont}}}$, ${\text {P}_{\text {mean}}}^3$, and $\log (\hbox {Vt}_{e})$, which all at least partially depend on the current ventilation mode or settings. The resulting estimation formula of the regression analysis is given by (1). The estimation method based on this result of the robust linear regression will be referenced as $\text {RLR-PaCO}_{2}$:

1 $$\begin{aligned} \begin{aligned} \text {PaCO}_{2} \ \hat{=} \ 31.8164&+ 0.8892 \cdot \text {etCO}_{2} - 0.0019 \cdot {{\text {O}_{2}}{^{\text {diff}}}^2} - 0.0854 \cdot {\%_{\text {spont}}}\\&+ 0.002 \cdot {\text {P}_{\text {mean}}}^3 - 5.2879 \cdot \log (\hbox {Vt}_{e}). \end{aligned} \end{aligned}$$

**Fig. 1:**
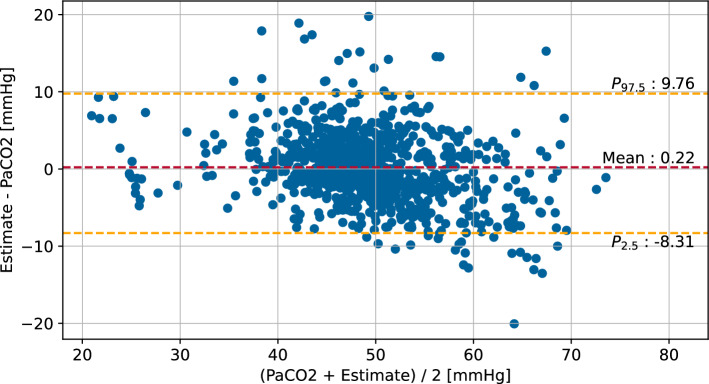
Bland–Altman plot of the $\text {RLR-PaCO}_{2}$ method and the arterial $\text {CO}_{2}$ measurement by blood gas analysis in the design population. Quantiles are used as limits of agreement due to non-normal distribution of differences [31]. Only simultaneous measurements with $\text {PaCO}_{2}$ $\le$ 75 mmHg and $\hbox {Vt}_{e}$ $\ge$ 12 mL are considered

The mean absolute error (MAE), standard error (SE), and standard deviation of differences (SD) of this new estimation equation in comparison to an estimation by unaltered $\text {etCO}_{2}$ are presented in Table 1. The comparison shows that $\text {PaCO}_{2}$ estimation by robust linear regression is more accurate than by unaltered $\text {etCO}_{2}$. Figure 1 shows the Bland–Altman plot of the $\text {PaCO}_{2}$ measurements by blood gas analysis and the estimation by the $\text {RLR-PaCO}_{2}$ method.

**Table 1:** Results of prediction of $\text {PaCO}_{2}$ by the $\text {RLR-PaCO}_{2}$ method compared to estimation of $\text {PaCO}_{2}$ by $\text {etCO}_{2}$ on the design population

Observed metric (*n* = 863)		$\text {etCO}_{2}$	$\text {RLR-PaCO}_{2}$
Mean absolute error (MAE) [mmHg]		6.80	3.54
Standard deviation (SD) [mmHg]		6.53	4.70
Standard error (SE) [mmHg]		0.22	0.16

Only simultaneous measurements with $\text {PaCO}_{2} \le {75\,}  {\text{mmHg}}$ and $\hbox {Vt}_{e} \ge {12\,} {\text{mL}}$ are considered. Results are rounded to two decimal digits

### Model evaluation

To examine the generalizability of $\text {RLR-PaCO}_{2}$, it was evaluated in independent animal trials, which were conducted after the used parameters for the estimation equation were identified and the according coefficients were parameterized. The evaluation population comprises 12 lambs with relevant characteristics shown in Table 2.

**Table 2:** Evaluation population characteristics and ventilation statistics

Clinical characteristics		**Animals **($\mathbf {n = 12}$)
Demographic characteristics		
Male [*n* ($\%$)]		9 (64.29)
Gestational age [days]		131.5 (131–133)
Weight [g]		3211 (3078–3480)
Ventilator parameters		
${\text {FiO}_{2}}$ [$\%$]		21 (21–27) [20–66]
Positive end-expiratory pressure [mbar]		7.7 (7.6–7.9) [0.4–8.0]
Mean airway pressure [mbar]		11.8 (10.8–12.7) [0.4–15.7]
Peak inspiratory pressure [mbar]		21.8 (18.8–24.1) [0.4–31.6]
Clinical parameters		
Respiratory rate [b/min]		53 (41–62) [23–126]
Spontaneous breathing percentage [%]		0 (0–0) [0–100]
Oxygen saturation [%]		94 (92–96) [68–100]
Minute volume [mL/min]		1058 (890–1248) [470–2185]
Expiratory tidal volume [mL]		21.2 (18.9–23.1) [11.4–37.4]
Blood gas		
pH [1]		7.33 (7.26–7.37) [6.95–7.61]
PaCO2 [mmHg]		49.7 (45.3–54.1) [25.2–102.0]
PaO2 [mmHg]		46.0 (40.0–52.3) [9.0–136.0]

Data are presented as median with interquartile range and range, except sex distribution

All performed and valid blood gas analyses are considered

Parameters are regarded only at the time around a blood gas analyses and averaged, as implemented in the evaluation. [b/min] stands for [breaths/min]

The proposed estimation method was integrated into the data recording tool of the animal trials with the goal to assess the performance of the estimation method in an online setting. Though it was not used for automation in the animal trials, the implementation should facilitate the usage in the future and test the online capabilities. To reduce the influence of outliers in the measured parameters, while still maintaining a low reaction delay, a 20 s averaging was introduced. The previous window of 2 min in the regression, as described in section "Regression approach", was overruled for two reasons. First, the needed, retrospective correspondence with a blood gas analysis was not given in the evaluation setting. Second, the reaction time to physiological changes should be kept to a minimum. In contrast to the proceeding in the regression analysis, this averaging furthermore only included measurements in advance of the estimation, such that no delay is created by waiting for data. Additionally, only $\hbox {Vt}_{e}$ measurements of 12 mL or above were used for the estimation to approach the inaccuracies described in section "Regression approach". The lack of such measurements led to an absence of estimates in the trial setting.

**Fig. 2:**
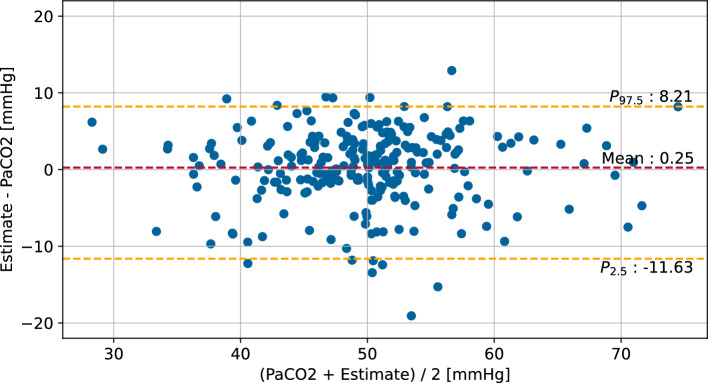
Bland–Altman plot of the $\text {RLR-PaCO}_{2}$ method and the arterial $\text {CO}_{2}$ measurement by blood gas analysis in the evaluation population. Quantiles are used as limits of agreement due to non-normal distribution of differences [31]. Only simultaneous measurements with $\text {PaCO}_{2}$ $\le$ 75 mmHg and $\hbox {Vt}_{e}$ $\ge$ 12 mL are considered

From the animal trials, 246 $\text {PaCO}_{2}$ measurements were used for the evaluation. Measurements with missing parameters or a $\text {PaCO}_{2}$ of more than 75 mmHg were excluded. The resulting Bland–Altman plot with respect to the measured $\text {PaCO}_{2}$ is shown in Fig. 2.

**Fig. 3:**
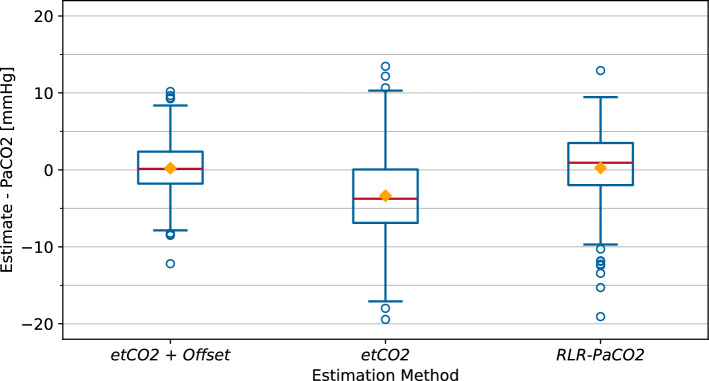
Deviation of the compared methods to the arterial $\text {CO}_{2}$ measured by blood gas analysis, visualized as boxplot. Mean values are represented by a diamond and the median as a horizontal line

The results should also be compared to current methods for $\text {PaCO}_{2}$ estimation to allow assessment of the results in this setting. Two different estimation methods were considered in this evaluation. First, the measured $\text {etCO}_{2}$ was corrected by an offset to generate an estimate ($\text {etCO}_{2}$ + offset). The offset is determined by the difference of $\text {etCO}_{2}$ with the $\text {PaCO}_{2}$ measured by blood gas analysis and changed as soon as the result is available. Therefore, this approach is premised on regular invasive adaptions. This estimation method is used in the conducted animal trials [30]. Second, the unaltered $\text {etCO}_{2}$ measurement was considered as estimation ($\text {etCO}_{2}$). A comparison of the deviation to the $\text {PaCO}_{2}$ of the three methods is shown in Fig. 3 as a boxplot.

**Table 3:** Results of the three considered estimation methods in comparison to $\text {PaCO}_{2}$ on the evaluation population without high $\text {PaCO}_{2}$ values, visualized as boxplot. Mean values are represented by a diamond and the median as a horizontal line

Observed metric (*n* = 246)		$\text {etCO}_{2}$+ offset	$\text {etCO}_{2}$	$\text {RLR}$-$\text {PaCO}_{2}$
Mean absolute error (MAE) [mmHg]		2.81	5.15	3.80
Standard deviation (SD) [mmHg]		3.66	5.44	4.92
Standard error (SE) [mmHg]		0.23	0.35	0.31

Only simultaneous measurements with $\text {PaCO}_{2} \le {75\,}\hbox {mmHg}$ and $\hbox {Vt}_{e} \ge {12\,}\hbox {mL}$ are considered. Results are rounded to two decimal digits

The $\text {RLR-PaCO}_{2}$ method exhibits a lower difference to the measured $\text {PaCO}_{2}$ than the unaltered $\text {etCO}_{2}$, while maintaining similar deviations from the mean difference. Table 3 shows that the $\text {RLR-PaCO}_{2}$ method exhibits a lower standard error and standard deviation of differences as the estimation by unaltered $\text {etCO}_{2}$ and reduces the mean absolute error by 1.35 mmHg. Both mentioned methods are less accurate than the offset-corrected $\text {etCO}_{2}$ estimation, as shown in Fig. 3 and Table 3. This estimation, nevertheless, relies on regular blood gas analyses. These constitute risks, especially in neonates, and are thus aimed to be reduced in their frequency.

**Fig. 4:**
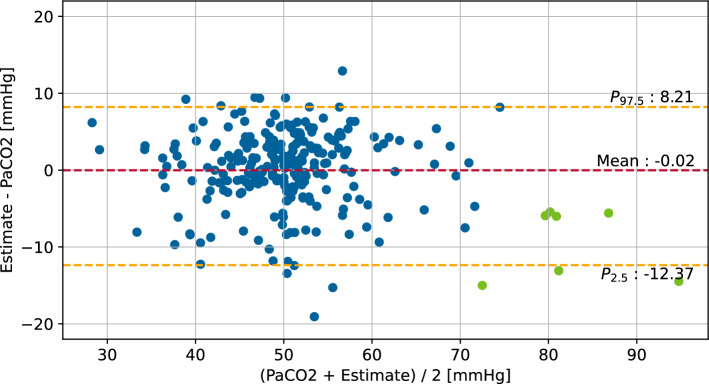
Bland–Altman plot of the $\text {RLR-PaCO}_{2}$ method and the arterial $\text {CO}_{2}$ measurement by blood gas analysis in the evaluation population. Quantiles are used as limits of agreement due to non-normal distribution of differences [31]. Points with $\text {PaCO}_{2} > {75\,} \hbox {mmHg}$ are shown in green. Only simultaneous measurements $\hbox {Vt}_{e}$ $\ge$ 12 mL are considered

The observation of an increasing $\text {etCO}_{2}$ inaccuracy in blood gas analyses with a $\text {PaCO}_{2} > {75\,} \hbox {mmHg}$, as described in section "Regression approach ", is also apparent in the evaluation population. When considering blood gas analyses with a $\text {PaCO}_{2}$ of more than 75 mmHg, seven additional blood gas analyses are included in the evaluation population. Table 4 shows a slight deterioration of all considered estimation methods in this setting with the strongest in unaltered $\text {etCO}_{2}$. The Bland–Altman plot of this extended evaluation population is shown in Fig. 4.

**Table 4:** Results of the three considered estimation methods in comparison to $\text {PaCO}_{2}$ on the evaluation population with high $\text {PaCO}_{2}$ values

Observed metric		$\text {etCO}_{2}$ + Offset	$\text {etCO}_{2}$	$\text {RLR}$-$\text {PaCO}_{2}$
Mean absolute error (MAE) [mmHg]		2.99	5.34	3.95
Standard deviation (SD) [mmHg]		4.03	5.60	5.15
Standard error (SE) [mmHg]		0.25	0.35	0.32

Only simultaneous measurements with $\hbox {Vt}_{e} \ge {12\,}\hbox {mL}$ are considered. Results are rounded to two decimal digits

## Discussion

In the present paper, we propose a new equation for estimation of $\text {PaCO}_{2}$ from $\text {etCO}_{2}$ by inclusion of physiological and ventilation parameters. For the development and evaluation, a preterm lamb model was used. We found that the proposed approach ($\text {RLR-PaCO}_{2}$) achieved a more precise estimation of $\text {PaCO}_{2}$ than unaltered $\text {etCO}_{2}$.

The $\text {RLR-PaCO}_{2}$ method achieves a mean absolute error of 3.80 mmHg, which is an enhancement of 26.21% in comparison to an estimation by unaltered $\text {etCO}_{2}$ measurements. Furthermore, it lowers the standard deviation of differences and standard error in the evaluation population of 12 neonatal lambs.

Only blood gas analyses with $\text {PaCO}_{2} \le {75\,}{\text{mmHg}}$ are regarded in this evaluation due to the increasing $\text {etCO}_{2}$ measurement deviations outside this range. When considering blood gas analyses with a $\text {PaCO}_{2}$ above 75 mmHg, the results in the evaluation population worsen only marginally. However, this is to be explained by the low proportion of such data points in the population. Figure 4 shows an underestimation of the $\text {PaCO}_{2}$ measurement by the $\text {RLR-PaCO}_{2}$ method in this span, supporting the observation in the design population.

The $\text {RLR-PaCO}_{2}$ method shows a substantial improvement in all considered metrics in the design population. This indicates a better estimation of $\text {PaCO}_{2}$ by the $\text {RLR-PaCO}_{2}$ method in comparison to unaltered $\text {etCO}_{2}$ measurements. In comparison to the offset-corrected $\text {etCO}_{2}$ measurement, $\text {RLR-PaCO}_{2}$ is inferior in all metrics. However, frequent blood gas analyses are necessary for correction of the offset, which is an essential disadvantage of this procedure. Furthermore, blood gas analyses are performed less often in clinical practice, which could potentially worsen the results of this method.

The highest initial correlation to the deviation of $\text {etCO}_{2}$ and $\text {PaCO}_{2}$ is present in ${{\text {O}_{2}}{^{\text {diff}}}^2}$. The variable ${{\text {O}_{2}}^{\text {diff}}}$ represents the relation between ${\text {SpO}_{2}}$ and ${\text {FiO}_{2}}$ in a simple subtraction. The presented ${{\text {O}_{2}}^{\text {diff}}}$ does not reflect the physiological non-linearity, e.g. originating in the oxygen saturation curve. This coherence and further non-invasive parameters should be examined, which could provide an enhancement of the estimation of $\text {PaCO}_{2}$ to $\text {etCO}_{2}$ difference.

In our evaluation population, 95% of estimates have a deviation between $-$11.63 mmHg to 8.21 mmHg in comparison to the $\text {PaCO}_{2}$ measured by blood gas analysis. Additionally, the estimates have a standard deviation of 4.92 mmHg. If this estimation dispersion is sufficient for clinical practice or the usage in applications like closed-loop control of $\text {PaCO}_{2}$ needs further evaluation. Also the averaging of the included parameters changed from training to evaluation population in order to achieve an online capable estimation formula with low delay. The effect of this change was not further examined. Nevertheless, in both the design and evaluation population, the $\text {RLR-PaCO}_{2}$ method lowers the mean absolute error and the standard deviation in comparison to an estimation by unaltered $\text {etCO}_{2}$.

### Limitations

The robust linear regression was performed on 51 animals in the same setting. All considered data were recorded on the first or second day of life. Due to the similar settings in the development and evaluation population, an overfitting to the data cannot be ruled out. Moreover, data from animal model might not be applicable to humans. The generalizability of the resulting estimation method should therefore be further investigated.

Observation showed that $\text {etCO}_{2}$ measurements for $\hbox {Vt}_{e} < {12\,}\hbox {mL}$ were imprecise for estimation of PaCO2. Common medical practice targets for $\hbox {Vt}_{e}$ are 4 mL/kg to 8 mL/kg, so the excluded volumes are typically used for patients weighing less than 3 kg. This, however, is the targeted patient group. The study data were obtained from late gestational preterm lambs of 130–135 days gestational age (term 147 days) weighing around 3 kg. The subjects of the animal model are thus larger than the targeted human patient population of preterm infants. This relatively mature lamb population was chosen to reduce the variability in this first series of animal experiments with a newly established controller-driven closed-loop ventilation for neonates. The size of the animals has also had some impact on the devices used for mechanical ventilation. A 4.5 mm inner diameter endotracheal tube (ET) was used and the particular anatomy of the lamb required a minimum length of the tube of 200 mm. The whole setup including ET, flow sensor and mainstream capnometry cuvette has an apparatus dead space of over 8 mL. The high apparatus dead space is a known difficulty in newborn capnometry [28]. In our opinion this is the most conclusive explanation for our observation, that reliable data were only available at tidal volumes of 12 mL or higher. We expect that this threshold is probably lower in smaller subjects who would be intubated with a smaller ET. However, we are also aware, that the dead space of components like the flow sensor or the mainstream cuvette would either need to be reduced, or that a sidestream measurement would need to be used instead.

Moreover, $\text {PaCO}_{2} > {75\,} \hbox {mmHg}$ were excluded due to increasing dispersion of the $\text {etCO}_{2}$ measurement. In a critical care setting this is a relevant disadvantage. Especially sick preterm infants with respiratory exhaustion and resulting hypercapnia cannot be targeted with this method. Furthermore, the significance of the introduced dead space by the $\text {etCO}_{2}$ sensor on human neonates is not targeted here and needs to be examined.

This study does only consider the accuracy and deviation of the estimation method in comparison to $\text {etCO}_{2}$. For the possible usage in applications like PCLC, properties like the robustness to perturbations need to be further analysed and methods for the continuous and reliable delivery of values, even in absence of input parameters or high $\text {PaCO}_{2}$ values, are to be developed and tested. For a more precise evaluation of practicability, data of neonatal patients should be used in a setting closer to everyday clinical practice and finally the usage for a closed-loop control of $\text {PaCO}_{2}$, as targeted in previous studies, is to be tested.

## Conclusion

We propose a new, non-invasive estimation method for $\text {PaCO}_{2}$ based on $\text {etCO}_{2}$ measurements as well as ventilator settings and measurements. The proposed estimation equation is generated using a robust linear regression with an iterative approach to the independent variable selection. For the regression and evaluation, two different sets of recordings from animal trials are used. Blood gas analyses with a $\text {PaCO}_{2}$ above 75 mmHg are excluded due to an increasing inaccuracy and variation of the $\text {etCO}_{2}$ measurement in this span. The resulting estimation method achieves a mean absolute error of 3.80 mmHg in the evaluation population. This lowers the mean absolute error of the unaltered $\text {etCO}_{2}$ measurement by 1.35 mmHg. An estimation by offset-corrected $\text {etCO}_{2}$ achieves a more accurate result. However, this estimation method achieves a mean absolute error of 2.81 mmHg, underlining the general complexity of continuous $\text {PaCO}_{2}$ estimation in the neonatal setting.

## Materials and methods

This study analysed secondary data originating from preterm lamb trials for the evaluation and refinement of a closed-loop control of $\text {PaCO}_{2}$ [30]. Only the pilot study was presented in the mentioned paper, leading to a much higher number of observed animals in this paper. These trials are based on the preterm ovine lung model. It is a well-established model for preterm pulmonary pathology and treatment [32, 33] and has gas exchange and acid base properties comparable to human beings [34]. All experiments were approved by the Dutch central commission for animal experiments (AVD10700202010347). The approach and procedures in this paper did not influence the animal trials in any way.

### Population

The design population consists of 59 mechanically ventilated, preterm lambs on the first day of life. Lambs were born by caesarian section at a gestational age of 130 to 135 days and intubated with a microcuff endotracheal tube (inner diameter 4.0 mm to 4.5 mm). In an ex utero intrapartum (EXIT) procedure, umbilical arterial and venous catheters were placed. Continuous analgosedation with midazolam and ketamine was applied in all lambs. In seven lambs endotracheal tube obstructions, right mainstem intubations, atelectases, and pneumothoraces were induced. None of the caused complications or ventilation conditions were assessed to infer with an estimation of $\text {PaCO}_{2}$. 8 out of 59 lambs were excluded because of insufficient data coverage due to technical issues ($n = 5$) or severe health complications resulting in a preliminary end of experiment ($n = 3$). No further exclusion criteria for animals were defined. Thus, 51 lambs were included in the design of the estimation method. Demographic characteristics as well as ventilator parameters, clinical parameters, and blood gas values of the considered population are given in Table 5.

**Table 5:** Design population characteristics and ventilation parameters

Clinical characteristics		Animals ($\mathbf {n = 51}$)
Demographic characteristics*		
Male [n ($\%$)]		22 (43.14)
Gestational age [days]		133 (132-134)
Weight [g]		3070 (2545-3472)
Ventilator parameters		
${\text {FiO}_{2}}$ [$\%$]		21 (21-30) [20-100]
Positive end-expiratory pressure [mbar]		7.7 (7.6-7.8) [0.9-9.715]
Mean airway pressure [mbar]		12.1 (11.3-13.9) [4.8-17.1]
Peak inspiratory pressure [mbar]		21.4 (18.1-24.6) [9.0-36.0]
Clinical parameters		
Respiratory rate [b/min]		61 (49-70) [18-101]
Spontaneous breathing percentage [%]		0 (0-0)[0-100]
Oxygen saturation [%]		95 (93-97) [68-100]
Minute volume [mL/min]		1123 (904-1423) [254-4758]
Expiratory tidal volume [mL]		19.8 (16.8-23.8) [12.1-84.9]
Blood gas		
pH [1]		7.27 (7.19-7.33) [6.61-7.94]
PaCO2 [mmHg]		49.9 (45.2-57.5) [17.0-130.0]
PaO2 [mmHg]		52.0 (44.0-64.0) [5.0-313.0]

Data are presented as median with interquartile range and range, except sex distribution

All performed and valid blood gas analyses are considered. Parameters are regarded only at the time around a blood gas analyses and averaged, as considered in the regression. [b/min] stands for [breaths/min]

*Sex data of four lambs and gestational age data of eight lambs are missing, one lamb was intersex

### Monitoring

The lambs were ventilated with a LeoniPlus respirator (Löwenstein Medical SE & Co. KG, Bad Ems, Germany) for neonates and children up to 30 kg with synchronized intermittent mandatory ventilation (SIMV). The real-time processing of measurements and the adjustment of ventilation parameters were enabled through modifications by the manufacturer. All available settings and measurements are recorded from the ventilator in frequencies from 0.5 Hz to 50 Hz, as provided by the interface of the manufacturer. The used measurements and settings for the extent of this paper are all recorded in a frequency of 0.5 Hz. No further processing of the provided ventilator data takes place. Capnometry was performed using a Masimo IRMA $\text {CO}_{2}$ proximal mainstream sensor (Masimo Corporation, Irvine, USA) connected to the ventilator. From this, the end-tidal $\text {CO}_{2}$ was calculated based on the maximum fraction of $\text {CO}_{2}$ in the last 4 s of the waveform without any further processing. The calculation is described in Appendix A. Additionally, the oxygen saturation (${\text {SpO}_{2}}$) was constantly measured using the integrated pulse oximeter module of the LeoniPlus ventilator, which uses Masimo SET^®^ technology (Masimo Corporation, Irvine, USA). Blood gas analyses were performed using an i-Stat 1 handheld blood gas analyser (Abbott Laboratories, Chicago, USA) at intervals of 15 min to 180 min, depending on the conducted experiments. Results were documented by hand.

### Regression approach

Appropriate variables for the estimation method were determined before the regression approach and are listed in Table 6. We introduced the variables ${{\text {O}_{2}}^{\text {diff}}}$ and ${{\text {O}_{2}}^{\text {quot}}}$ as two straightforward measures for the relation between ${\text {FiO}_{2}}$ and ${\text {SpO}_{2}}$. While the ${\text {SpO}_{2}}$ to ${\text {FiO}_{2}}$ ratio is discussed as a non-invasive measure with relation to the Horowitz quotient [35–37], the difference of these parameters was introduced without known evidence, but as possible indicator of ventilation efficiency. For all variables, the square and cube were additionally included. The logarithm was included in variables with none or infrequent zero values.

**Table 6:** Considered variables for the regression analysis

Name		Description
${\text {etCO}_{2}}$ [mmHg]		Calculated end-tidal $\text {CO}_{2}$ from capnometry
${\text {FiO}_{2}}$ [$\%$]		Set fraction of inspired $\hbox {O}_{2}$
PEEP [mbar]		Applied positive end-expiratory pressure
$P_{\hbox {peak}}$ [mbar]		Applied peak pressure
$p_{\hbox {mean}}$ [mbar]		Measured mean applied pressure
*f* [b/min]		Measured breath frequency
$f_{\hbox {Spont}}$ [b/min]		Measured spontaneous breath frequency, including triggered breaths
$\%_{\hbox {Spont}}$ [%]		Measured percentage of spontaneous breathing share, including triggered breaths
MV [mL/min]		Measured minute volume
${\text {SpO}_{2}}$ [%]		Measured oxygen saturation
${\hbox {Vt}_{i}}$ [mL]		Measured inspiration volume
${\hbox {Vt}_{e}}$ [mL]		Measured expiration volume
Compl [mL/mbar]		Measured dynamic compliance
Leak [%]		Measured leakage
Resist [mbar/L/s]		Measured resistance
${{\text {O}_{2}}^{\text {diff}}}$ [%]		Difference between set fraction of inspired oxygen and measured oxygen saturation (${\text {FiO}_{2}}$ - ${\text {SpO}_{2}}$)
${{\text {O}_{2}}^{\text {quot}}}$ [1]		Ratio of measured oxygen saturation and fraction of inspired oxygen (${\text {SpO}_{2}}$ / ${\text {FiO}_{2}}$)

Here, [b/min] stands for [breaths/min]. All variables are recorded at a frequency of 0.5 Hz. $\text {etCO}_{2}$ is calculated at a frequency of 0.25 Hz

Blood gas analyses were included, if all considered variables were available at the time of blood sampling. Blood gas analyses with a $\text {PaCO}_{2}$ above 75 mmHg were excluded from regression analysis. This was justified by an increasing underestimation by the unaltered $\text {etCO}_{2}$ measurements and an enhancing variation in this span. The inclusion of these datapoints would lead to a worsening of the entire estimation method. On exclusion of the 69 affected data points, the mean absolute error of the estimation by $\text {etCO}_{2}$ decreases by 16.14% and the standard deviation by 24.25%. On this basis, an accurate estimation of $\text {CO}_{2}$ above 75 mmHg is not targeted with this method. A targeted set-point for the $\text {PaCO}_{2}$ of above 75 mmHg is very rare in clinical practice. The presence of $\text {PaCO}_{2}$ values in this range therefore commonly results in the general need to intensify ventilation. A precise estimation of $\text {PaCO}_{2}$ values in this range is therefore considered as less relevant for clinical practice and most applications of the proposed estimation method. Furthermore, $\text {PaCO}_{2}$ measurements with a simultaneous expiration volume of less than 12 mL were excluded, as the end-tidal $\text {CO}_{2}$ measurement was observed to become imprecise below this threshold.

For all included blood gas analyses, the considered variables are averaged over a 2-min window with its centre at the minute of the blood gas analysis. This is done because only the minute and not the exact time of the blood gas analysis is documented. A window of 2 min is considered suitable, as the exact time of the blood gas analysis will be included in this interval. Moreover, the effect of outliers is reduced while only minor physiological changes are expected during this time in most cases.

The regression analysis is implemented using the programming language R (Version 4.2.1, The R Foundation for Statistical Computing, Vienna, Austria). The considered data showed a tendency in the difference between $\text {etCO}_{2}$ and $\text {PaCO}_{2}$ with the exception of some outliers. Although the outliers are relevant measurements, we aimed to achieve a high estimation accuracy on the majority of the data, while accepting a higher deviation in single outliers. Least squares linear regression is known to be prone to such points and therewith loses in accuracy on the majority of data points [38]. Thus, a robust linear regression is used, which is enabled by the MASS package (Version 7.3-57, Brian Ripley). The variables included in the final model are determined in an iterative fashion. This approach assumes, that the variable with the highest correlation to the remaining estimation deviation has the highest potential to explain this deviation. If this correlation is existent in general, the inclusion in the regression should lead to a more precise estimation. Thus, in every iteration, the variable with the highest linear correlation (Pearson coefficient) to the difference between current estimate and $\text {PaCO}_{2}$ measurement is included in the regression. With the newly included variable, a further robust linear regression is performed to calculate the new deviations in the next iteration. This process is terminated once no variable exhibits a correlation above 0.2, as no substantial enhancement of the estimation is observable beyond this point.

## Data Availability

The datasets analysed during the current study are not publicly available due to a cooperation agreement with an industrial partner but are available at doi.org/21.11102/7a0dcedf-f6c8-4276-83da-9aadbb514f53 on reasonable request.

## Appendix A: End-tidal $\hbox {CO}_{2}$ calculation

The $\text {etCO}_{2}$ is calculated every four seconds by (2):

2 $$\begin{aligned} \text {etCO}_{2} = (P_{\text {amb}} + {\text {PEEP}}) \cdot (\text {CO}_{2}^{\text {max}} / 100) \cdot 0.750062. \end{aligned}$$

Here, $P_{\text {amb}}$ is the measured ambient pressure and $\text {CO}_{2}^{\text {max}}$ the highest fraction of $\text {CO}_{2}$ in the last 4 s in percent. 0.750062 is the conversion factor from mbar to mmHg, as $P_{\text {amb}}$ and $\text {PEEP}$ are measured in mbar.
